# Systemic Sclerosis With Cardiac Manifestations: A Case Report

**DOI:** 10.7759/cureus.64952

**Published:** 2024-07-19

**Authors:** Jamie Sison, Jamis Gouge, Ellen Pikus

**Affiliations:** 1 Internal Medicine, Atrium Health Navicent The Medical Center, Macon, USA

**Keywords:** biventricular conduction delay, right bundle branch block, left anterior fascicular block, systemic scleroderma, systemic sclerosis (ssc

## Abstract

Systemic sclerosis (SSc), or scleroderma, is a multisystem disease process that can result in significant end-organ damage if left undiagnosed or untreated. While some manifestations are well-known and widely researched, other presentations of SSc, including the presentation of our patient, require further investigation. Though many non-pulmonary and non-dermatologic manifestations lack widespread recognition, such presentations are important to recognize clinically in order to adequately investigate and appropriately treat. Fibrotic changes affect not only the skin but also the myocardial conduction system which can result in chronic systolic heart failure and significant conduction delays. This report is a case of newly diagnosed scleroderma that presented with worsening dyspnea and activity intolerance who was discovered to have new onset prolonged PR interval, right bundle branch block, and left anterior fascicular block. After a comprehensive workup, the patient was diagnosed with scleroderma and underwent treatment by a multidisciplinary team.

## Introduction

Systemic sclerosis (SSc) is a chronic and complex autoimmune disorder characterized by progressive fibrosis and vasculopathy [1]. This pathologic process results in both thickening and hardening of the skin, also known as scleroderma, dysfunction of small blood vessels throughout the body [2], and Raynaud's phenomenon, which presents as digital color change in response to cold or stress. The exact etiology of SSc remains unknown, though a complex interaction between genetic predisposition and environmental factors likely contribute to the development of this disease [3]. Clinically, SSc presents as a wide variety of manifestations, with two main subtypes: limited cutaneous SSc (lcSSc) and diffuse cutaneous SSc (dcSSc) [4]. These subtypes are classified based on the extent of skin involvement, but systemic sclerosis can involve multiple organ systems, including the gastrointestinal tract, lungs, heart, and kidneys. This systemic involvement leads to a range of complications such as pulmonary hypertension, interstitial lung disease, gastrointestinal dysmotility, and renal crisis. Our case presents a middle-aged male who presented in what was assumed to be an acute exacerbation of new-onset heart failure with reduced ejection fraction (HFrEF) and was ultimately diagnosed with scleroderma.

## Case presentation

A 57-year-old male with chronic bilateral lower extremity edema of two to three years duration and lymphedema presented to the emergency department with worsening dyspnea and activity intolerance of two to three months duration. His past medical history was significant for chronic kidney disease (CKD) stage 4 and hypertension (HTN), and he had been recently hospitalized for hypertensive urgency that improved with significant diuresis and medication adjustment. The patient noted the accompanying shortness of breath began about the time of increased leg swelling approximately two to three months prior to admission that worsened with activity. The patient reported a fall while showering after feeling dizzy which prompted him to present to the emergency room secondary to increased shortness of breath, decreased ambulation, and increased leg swelling. He denied any chest pain, nausea or vomiting, or diarrhea though did report a subjective 30-pound weight loss over the last three months. Emergency workup was significant for N-terminal pro-brain natriuretic peptide (NT-proBNP) elevated at 12,086 pg/mL and mildly elevated high sensitivity troponin at 153 ng/L (Table 1).

**Table 1:** Cardiac Markers NT-proBNP: N-terminal pro-brain natriuretic peptide, HS: high sensitivity

Cardiac Markers	Patient Values	Reference Range
NT-pro BNP	12086 pg/mL (H)	0-210 pg/mL
Troponin HS	153 ng/L (H)	<35 ng/L

**Figure 1:**
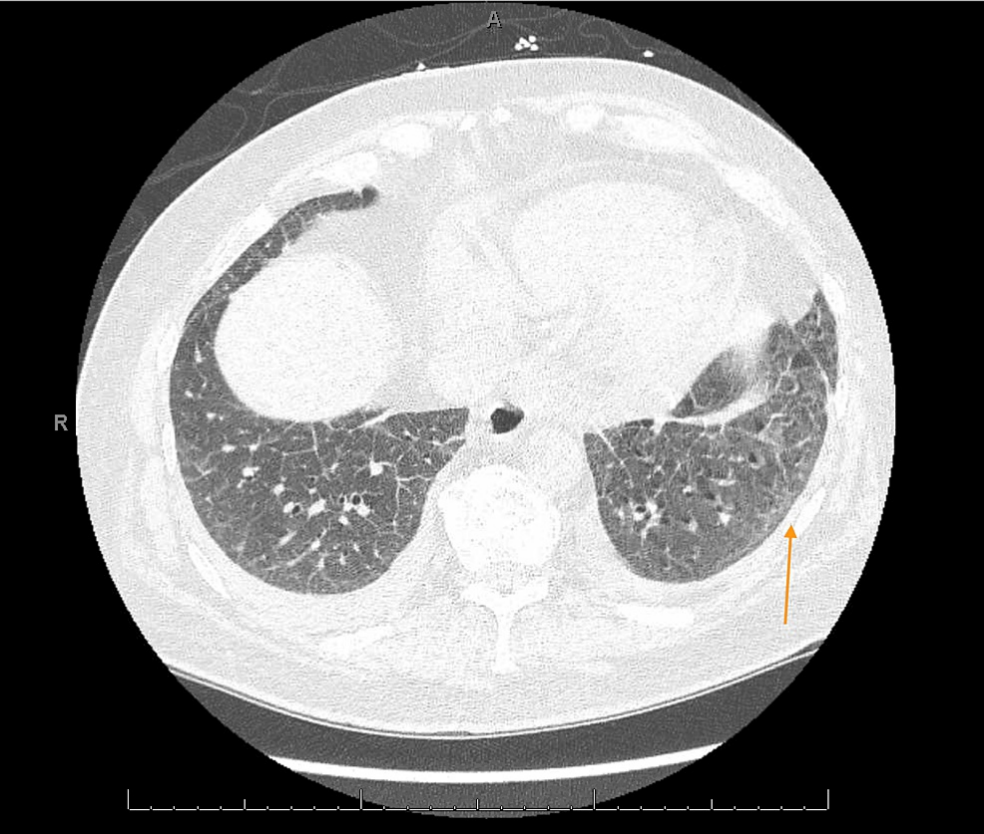
High Resolution CT Chest with Mild Ground Glass Opacities, Left Middle Lobe

**Figure 2:**
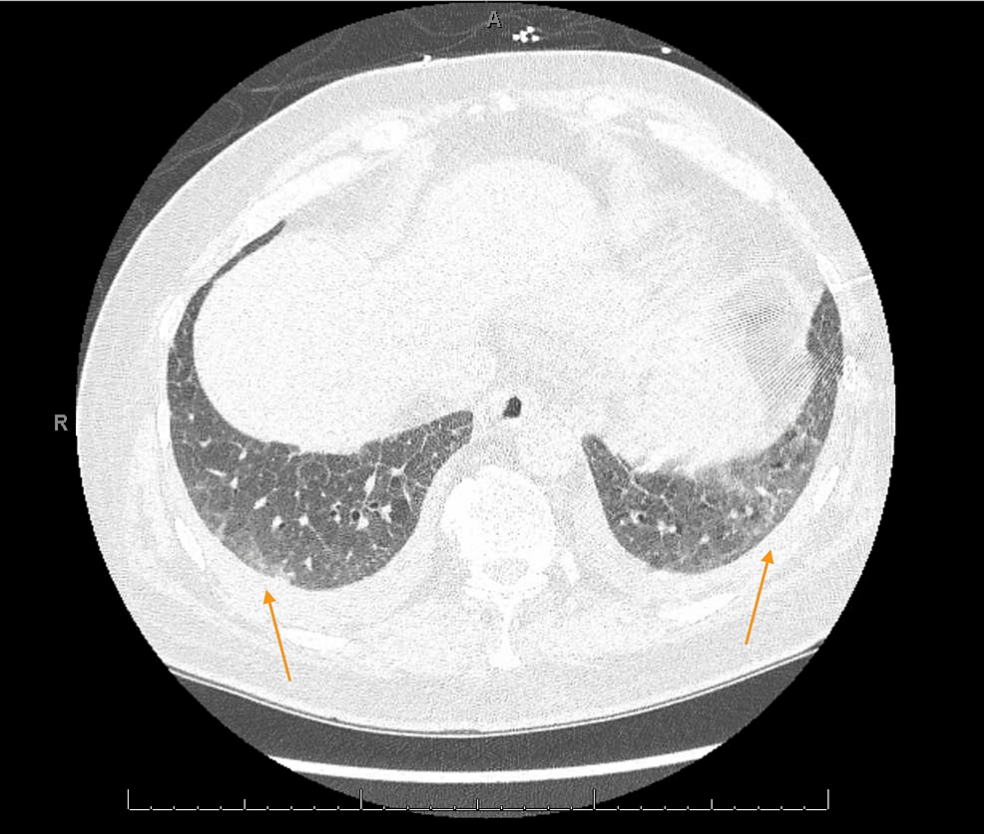
High Resolution CT Chest with Mild Bilateral Ground Glass Opacities, Middle Lobes

**Figure 3:**
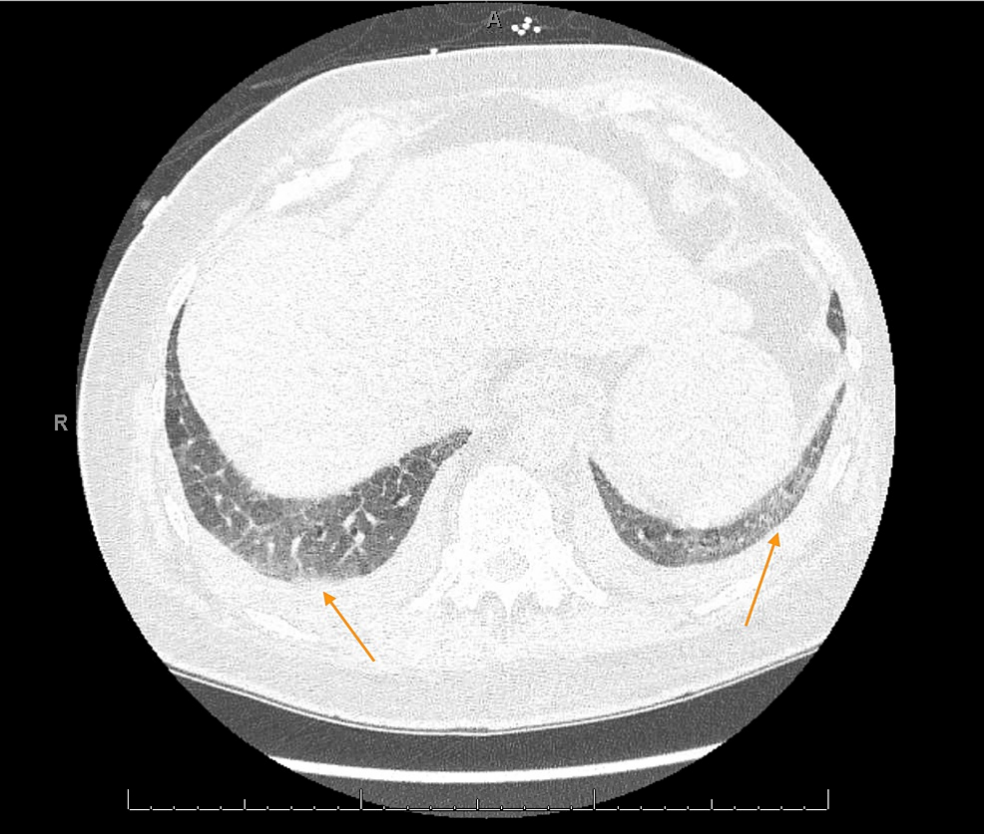
High Resolution CT Chest with Mild Bilateral Ground Glass Opacities, Lower Lobes

**Figure 4:**
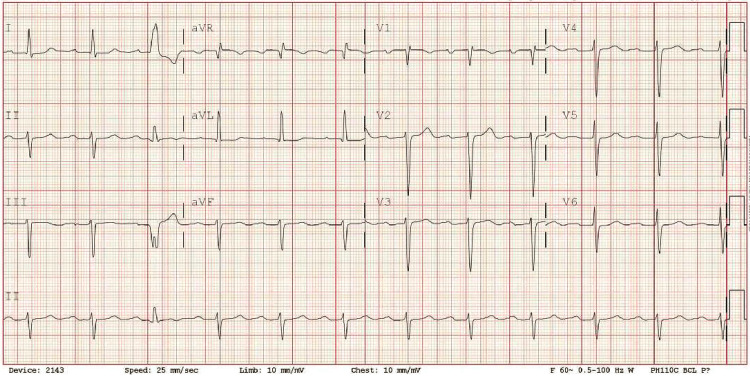
EKG: Prolonged PR interval, Incomplete RBBB, and Incomplete LAFB RBBB: right bundle branch block, LAFB: left anterior fascicular block

The patient was initially thought to be in an acute exacerbation of new-onset heart failure however transthoracic echocardiography (ECHO) revealed low normal left ventricular (LV) systolic function with ejection fraction (EF) of 50-55% and normal right ventricular size and function (Video 1). On physical exam, he was noted to have dactylitis, telangiectasias, and Raynaud phenomenon. Further workup was significant for positive antinuclear antibodies (ANA) and scleroderma SCL-70 antibodies. Due to strong clinical suspicion of scleroderma, high-resolution CT chest was done showing nonspecific ground glass opacities without honeycombing which, along with significant dyspnea, could be considered early signs of pulmonary involvement (Figures 1-3). During admission, the patient developed runs of nonsustained ventricular tachycardia on telemetry and complained of palpitations. Electrocardiograms (EKGs) were consistent with a prolonged PR interval, incomplete left anterior fascicular block (LAFB), and incomplete right bundle branch block (RBBB) (Figure 4) leading us to infer that due to multiple conduction delays, scleroderma can considerably affect the cardiac conduction system. Cardiology and electrophysiology were consulted for assistance with inpatient management of new-onset arrhythmias. The patient’s symptoms of dyspnea and dizziness improved with diuresis by intravenous furosemide over several days to where he no longer required supplemental oxygen support then transitioned to oral furosemide. Ultimately, the patient was discharged medically stable and improved to baseline.

**Video 1:** Transthoracic Echocardiography (TTE)

The patient was discharged on furosemide 40mg oral daily, metoprolol 25mg bid per cardiology recommendations, nifedipine XR 30mg daily, and the patient was continued on losartan 100mg daily per nephrology recommendations for renoprotective effects. Prednisone was not prescribed to prevent precipitation of sclerodermal renal crisis. Patient was also found to be hyperphosphatemic (mild 4.9) and with secondary hyperparathyroidism thus was started on cholecalciferol daily.

He was referred to rheumatology and pulmonology outpatient along with close follow-up with his primary care physician, cardiology, and electrophysiology.

## Discussion

It is well known that SSc is associated with a wide range of multi-system involvement and specifically, a spectrum of cardiac involvement. Cardiac manifestations in SSc are varied and can occur due to primary mechanisms such as fibrosis, vascular dysfunction, intrinsic cardiomyocyte abnormalities, or secondary mechanisms triggered by complications like pulmonary arterial hypertension (PAH), interstitial lung disease (ILD), cardiac inflammation, or scleroderma renal crisis [5]. The pathogenesis of primary cardiac involvement in SSc is characterized by a cascade of events initiated by recurrent coronary microvascular ischemia and myocardial inflammation. Repetitive episodes of limited blood flow to tiny coronary vessels (microvascular ischemia) and inflammation within the heart muscle (myocardial inflammation) culminate in cell death due to lack of oxygen (ischemic necrosis), further tissue injury during reperfusion leads to progressive scarring of the heart muscle (myocardial fibrosis). In this case, the patient had new onset palpitations and ventricular tachycardia as well as significant EKG findings [5]. These clinical manifestations, along with objective EKG and CT findings suggest that this patient the possibility of cardiomyopathy due to scleroderma, even with a normal EF on ECHO and that this patient has a combination of primary and secondary mechanisms contributing to cardiac disease. The patient had hypercalcemia and notable coronary artery calcifications on CT, suggestive of fibrosis and vascular dysfunction. The patient’s chronic lymphedema and CKD 4 may represent multisystem vascular disease. On chest x-ray, there were ​​increased interstitial markings in the infrahilar regions bilaterally and ground glass opacities in the lungs signifying inflammatory interstitial changes likely indicating the beginnings of interstitial lung disease.

Though cardiac involvement of scleroderma is typically represented by heart failure, it is crucial to remember that arrhythmias and conduction abnormalities also represent significant disease burden. Myocardial fibrosis, a hallmark feature of the SSc, disrupts the heart's electrical conductivity. As such, arrhythmias, a serious complication of SSc, are linked to roughly 6% of deaths in patients [6]. Literature review shows that patients with scleroderma frequently experienced premature contractions of both the atria and ventricles, with RBBB noted as the most common conduction abnormality. Importantly, elevated levels of NT-pro BNP, a marker of heart stress, were also associated with a higher frequency of various arrhythmias, including RBBB [6]. Specifically, a study by Follansbee et al. linked specific arrhythmias with either normal or abnormal EF. RBBB and isolated LAFB were both associated with normal EF, whereas left bundle branch block (LBBB) and bifascicular block (combination of RBBB and LAFB) were linked with abnormal left ventricular function [7]. Follansbee et al.’s research suggests that, based on the conduction delays noted in this patient, without treatment, he would likely develop worsening LVEF and ultimately HFrEF.

## Conclusions

Scleroderma can affect many parts of the body, but cardiac involvement is less well-known than some, more widely researched and written complications. SSc can manifest in various ways, and cardiac involvement in and of itself can present with a wide range of symptoms. This case highlights cardiac involvement in scleroderma and even some less commonly seen cardiac manifestations of SSc that are important to recognize and treat appropriately to prevent progression of disease and address symptom management. Scleroderma requires a multidisciplinary approach and collaboration between rheumatologists, cardiologists, electrophysiologists, and pulmonologists for optimal management. This case report on Scleroderma and cardiac manifestations can hopefully contribute to improved understanding, earlier diagnosis, better treatment strategies, and ultimately, improved patient outcomes.
